# Supplementary material for: Running and tumbling with E. coli in polymeric solutions
Source: Sci Rep. 2015 Oct 28;5:15761. doi: 10.1038/srep15761 (PMC4938119; doi:10.1038/srep15761)
Supplement: Supplementary Information [file srep15761-s1.pdf]

Running and tumbling with *E. coli* in polymeric solutions

A. E. Patteson[1], A. Gopinath[1,2], M. Goulian[3], and P. E. Arratia[1]

[1] *Department of Mechanical Engineering & Applied Mechanics, University of Pennsylvania, Philadelphia, PA 19104*
[2] *School of Engineering, University of California Merced, Merced, CA 95343*
[3] *Department of Biology, University of Pennsylvania, Philadelphia, PA 19104*

I. Rheological characterization of solutions

A. Shear viscosity and elasticity of CMC solutions

The shear viscosity of the carboxymethyl cellulose solutions is measured with a cone-and-plate rheometer, over a range of shear rates 10 to 1000 s^{-1}. For dilute suspensions (c = 10 ppm), the fluid is nearly Newtonian (c.f Fig 1a). However, at higher concentrations, the solutions are shear thinning. We define an effective viscosity experienced by the bacterial cell as the average shear viscosity over the shear rates of 10-100 s^{-1}. This range of shear rates was chosen since it corresponds to *E. coli* flagellar bundle rates (50-100 s^{-1}) [1, 2]. As shown in Fig. 1, the viscosity increases from 1.0 to 20.0 mPa s with polymer concentration for polymer molecular weight 9.0 x 10^4, 2.5 x 10^5, and 7.0 x 10^5.

At low concentrations, the shear viscosity is nearly constant with shear rate. At higher concentrations, particular for MW = 7.0x10^5, the shear viscosity begins to decrease with increasing shear rate (Fig. 1a). The shear-thinning viscosity η can be described by a model power law fluid, such that $\eta = K\dot{\gamma}^{n-1}$, where n is the shear-thinning index. At the highest concentration (c = 500 ppm, MW = 7.0x10^5), the shear-thinning index reaches a minimum of 0.70, as shown in Table I.

The fluid elasticity is quantified by measuring the relaxation time, λ of the CMC solutions (MW 7.0x10^5). This time scale is determined via a creep recovery test in a microfluidic device [3]. We find that the relaxation times increase from 0.02 seconds to 2.0 seconds as the polymer concentration varies from 200 to 4000 ppm, as shown in Fig. 2. The relaxation times we measure in the microfluidic device are qualitatively consistent with measurements made independently in a macroscopic cone-and-plate rheometer [4] and provides a measure of the elasticity in the highest concentrated CMC solutions used *with E. coli* (c = 225, 500 ppm).

c (ppm)	$\mu\ (mPa\ \cdot s)$	n	$\lambda\ (ms)$
0	0.97	-	-
10	1.38	.95	-
35	2.92	.84	-
60	4.51	.77	-
100	6.81	.73	-
225	11.8	.72	23.0
500	18.8	.70	49.5

TABLE I: Rheological properties of CMC (MW = 7.0 x 10^5) solutions, including shear viscosity μ, shear thinning index n, and relaxation time λ.

B. XG Rheology

As mentioned in the previous section, the relaxation times λ are determined via a creep recovery test in a in-house microfluidic device. We find that for the highest XG concentration (c = 300 ppm), λ is approximately 44 ms, as shown in Fig. 2.

The shear viscosities of XG solutions are measure in a cone and plate rheometer. As shown in Fig. 3, the viscosity significantly shear thins, particularly in the region of interest, $\dot{\gamma}$ =10-100 s^{-1}. At the highest concentration, the shear-thinning index n reaches a minimum of 0.50 (Table 2).

c (ppm)	n
10	1.00
30	0.92
60	0.76
100	0.64
180	0.57
300	0.51

TABLE 2: Shear thinning index of XG solutions.

C. Rheology of PEG solutions

The shear viscosities of PEG solutions are measured with a cone-and-plate rheometer. For the range of concentrations used (c = 1.3-10% by weight), the shear viscosity increases from 2 to 30 mPa· s, as shown in Fig. 4. Furthermore, shear viscosity is nearly constant with shear rate, indicating that PEG solutions provide a model Newtonian fluid.

II. MSD for model run-and-tumble cells

To obtain a relationship between the translational diffusivity and the mean square displacement, we consider a very simple model cell that undergoes an idealized, continuously diffusive process involving a sequence of runs and tumbles. Unlike a real bacterial cell, however we assume that the tumble angles are arbitrary and drawn completely randomly. We begin by choosing an initial position and orientation of the cell at time. The cell is located at **r** at time *t* and oriented with an angle θ moving at constant speed v but whose direction of motion is affected by the previously alluded to random rotations. The position and orientation of the cell follows then the equations ${d\boldsymbol{r}}/{dt} = \boldsymbol{t}v$, and ${d\theta}/{dt} = \eta(t)$. Here, $\eta(t)$ is a zero-mean, delta-correlated Gaussian random variable such that $\langle \eta(t)\eta(t') \rangle = 2D_R\delta(t-t')$ and $\boldsymbol{t}$ is the direction of the run which is along the instantaneous, local tangent to the trajectory.

Using standard techniques, $\Delta\theta(t) = \theta(d + \Delta t) - \theta(t) = \int_t^{t+\Delta t} \eta(t')\, dt', \langle\Delta\theta(t)\rangle = 0$ and $\langle\Delta\theta(t)\Delta\theta(t')\rangle = 2D_R \min(t, t')$. Application of the central limit theorem shows that $\Delta\theta$ has zero mean, and is distributed following a Gaussian profile. Thus the pdf (probability density function), ψ is given by $\psi(t, \Delta\theta) = (\frac{1}{4\pi t D_R})^{\frac{1}{2}} \exp(-\frac{\Delta\theta^2}{4\pi t D_R})$ which may then be readily used to calculate averages. The mean square displacement (MSD), assuming the run velocity v is constant, is obtained by evaluating

$$< |\boldsymbol{r}(t + \Delta t) - \boldsymbol{r}(t)|^2 > = v^2 \int_t^{t+\Delta t} dt' \int_t^{t+\Delta t} < \cos[\theta(t') - \theta(t'')] > dt''$$

which yields on simplification

$$MSD = 2\left(\frac{v^2}{D_R}\right)\left(t - \frac{1-e^{-\Delta t\, D_R}}{D_R}\right). \quad \text{(SI-1)}$$

One can consider asymptotic approximations to the complete expression for short times (A_1) and long times (A_2) by expanding (SI-1) in terms of the parameter ($\Delta t D_R$). When this dimensionless parameter is small, we find using a Maclaurin series expansion that $A_1 \sim v^2(\Delta t)^2$. In the complementary, long time limit, we obtain the asymptotic expression $A_2 \sim 2\left(\frac{v^2}{D_R}\right)\Delta t$. The effective translational diffusivity is obtained by rewriting (SI-1) by introducing an average run time τ, that characterizes the time for the MSD to transition from ballistic to diffusive behavior. The complete form of the MSD then is

$$MSD = 4D_T\Delta t\left(1 - \frac{\tau}{\Delta t}\left[1 - e^{-\Delta t/\tau}\right]\right) \quad \text{(SI-3)}$$

For long times, when A_2 dominates A_1 that is when the diffusive part due to active tumbling and reorientation dominates the ballistic rigid segment of the trajectory due to directed propulsion, the bacteria trajectory may be characterized by an effective translational diffusion coefficient.

Previous studies have used an approximation to this expression that we employ in the main text. While the two forms differ in the functionality of the exponentially decaying term, they have similar asymptotic short and long time behaviors. The main difference between the two arises when $\Delta t \sim\tau$.

III. MSD Crossover time increases with polymer concentration

For *E. coli*, the dynamics of its entire trajectory can be captured using the relationship $MSD(\Delta t) = 4D_t\Delta t(1 - e^{-\Delta t/\tau})$, where τ is a typical crossover time marking the transition from ballistic and diffusive manner. The crossover time is related to the mean run time of the ballistic runs τ_R. The MSD is proportional to $4D_T(\Delta t)^2/\tau$ for $\Delta t \ll \tau_R$ and to $4D_T\Delta t$ for $\Delta t \gg \tau_R$. By fitting the MSD data in Fig. 3(a) to this relation, we find that the crossover time τ increases with polymer concentration from 0.9 to 4.8 s, as shown in Fig. 5.

IV. *E. coli* Rotational Diffusivity and Mean Run Time

The rotational diffusion D_R for a run-and-tumble particle depends on the mean tumble angle α and the mean run time τ_R as $D_R = (1 - \cos\alpha)/\tau_R$ [5]. The tumble angles are defined by the angular change in direction from run to run, as shown in Fig. 6a. Averaging over multiple tumbles and multiple cells, we find the mean tumble angle remains nearly constant with polymer concentration Fig. 6b. The tumble angle depends on the effective torque acting on the cell body that rotates it about an axis different from the direction of motion– the rotation being due to the fact that the flagella are unbundled [1]. Specifically, the tumble angle is proportional to this “tumble” torque τ_t and the tumble time. The tumble torque, τ_t is proportional to the fluid viscosity. In our experiments, the fluid viscosity varies by a factor of about 20. Thus, we expect the tumble torque to increase correspondingly by a factor of approximately 20 as well. However, this effect combined with the simultaneously observed increased in mean tumble times (Fig. 4b and 4e in main text) results in a nearly constant mean tumble angle. A cell attempting to tumble in polymer solutions is successful and can thus change direction by the same amount as in a fluid without polymer. This suggests that the change in rotational diffusion of cells (Fig. 4c in main text) is primarily due to the changes in run time and not changes in tumble angle.

V. Suppression of Wobbling with Molecular Weight

Figure 7a shows the mean cell velocity $\langle v \rangle$ as a function of fluid viscosity for CMC solutions of different MW and a XG solution. While $\langle v \rangle$ increases with μ for the highest molecular weight CMC and XG solutions, the relative enhancement in $\langle v \rangle$ diminishes as the CMC molecular weight (and thus elasticity) decreases. This is evident if one selects a specific viscosity (μ = 11 mPa · s, shaded in Fig. 7a, where $\langle v \rangle$ clearly decreases with CMC molecular weight. The increase in $\langle v \rangle$ with CMC molecular weight (MW) is consistent with the simultaneous decrease in wobbling $\langle \sigma(\phi) \rangle$ with MW, shown in Fig. 7b for μ = 11 mPa · s. We note that *E. coli* swimming in the highest MW CMC and XG solutions show effectively the same degree of wobbling $\langle \sigma(\phi) \rangle$ (and $\langle v \rangle$) even though their power law indexes are quite different, which strongly indicates that shear-thinning viscosity effects are not important.

VI. Polymer dynamics due to flow generated by tethered cells

We quantify polymer extension by measuring an effective length of the polymer molecule, ℓ, defined to be the maximum distance along two points of the cell's contour. This allows us to quantify the polymer extension in terms of a single dimensionless function -- the distribution of ℓ/ℓ_c -- where ℓ_c is the contour length of (typically 22.0 μm for our DNA polymer) [6]. Note that this is *not* the same as the end-to-end distance and so statistics of this normalized length yield order of magnitude estimates.

For weak flow fields, an estimate of the fluid force resulting in polymer stretching can be estimated by examining the distribution of ℓ/ℓ_c over a time for which the polymer feels the constant flow gradients. While the actual flow field is time dependent, one may assume that sampling and reorientations are sufficiently quick. In our case, this would correspond to sampling the lengths when the polymer is at the same distance from the tethered cell. For ease of analysis, we

assume that the statistical properties of the end-to-end distance are comparable to that of ℓ/ℓ_c of the DNA blob. Hence we now study the shift in the distribution function characterizing the end-to-end distance as a function of extensional forces generated as the DNA polymer samples the local flow field.

The DNA configuration can typically be modeled as a self-avoiding walk (SAW) in two-dimensions. The distribution of end-to-end distance, x, under thermal fluctuations alone follows $PDF(x) = ax^{1+\sigma}e^{-bx^{\delta}}$ with scaling exponents given by $\sigma = 0.44$ and $\delta = 4$ [7, 8]. The constant a is determined by the normalization condition. The constant b is related to the mean end-to-end distance and depends on the number of effectively independent chains constituting the polymer chain, N, and an effective segment length ($r_0 = 50$ nm [6]), and is given by $b = (r_0 N^{3/4})^{-\delta}$ where the contour length $\ell_c = r_0 N$. Rewritten explicitly in terms of the contour length, we get in the absence of imposed forces the equilibrium distribution

$$PDF(x/\ell_c) = {\ell_c}^{1+\sigma} a(\frac{x}{\ell_c})^{1+\sigma} e^{-b^*(\frac{x}{\ell_c})^{\delta}} \text{ where } b^* = b\,(\ell_c)^{\delta} = r_0^{\delta} N^{\delta/4} \quad \text{(SI-3)}$$

When a constant stretching (elongational) force *F* is applied to the polymer, the distribution function of end-to-end distances shifts. The distribution shifts accordingly to accommodate entropic effects [9] and attains the form:

$$PDF(x/\ell_c) = {\ell_c}^{1+\sigma} a(\frac{x}{\ell_c})^{1+\sigma} e^{-b^*(\frac{x}{\ell_c})^{\delta}} e^{\frac{\ell_c F(\frac{x}{\ell_c})}{k_B T}}. \quad \text{(SI-4)}$$

We next quantify the PDF of the scaled stretch near the cell (at a radial distance 5 μm) and the PDF of the DNA in the absence of a cell both shown in Fig. 8. Modeling the polymer in the absence of cells using equation (SI-3), and fixing $\sigma = 0.44$ and $\delta = 4$, we obtain *b** = 3100. By fixing $\sigma = 0.44$, $\delta = 4$ and *b** = 3100, we next fit the data in Fig. 6b to (SI-4) and obtain *F* = *4.5* fN as an estimate of the effective fluid flow induced extensional force stretching the polymer, as shown in Fig. 8.

VII. Estimation of Weissenberg Numbers

A. Weissenberg numbers for freely swimming cells

The Weissenberg number W*i* is defined here as the product of the fluid relaxation time λ and the characteristic frequency of the swimmer *f*, where *f* is the rotation rate of the *E. coli* bundle. Thus, $Wi = 2\pi f\lambda$. The relaxation times are estimated from previous measurements of CMC solutions in a macroscopic cone-and-plate rheometer [7] using a simple extrapolation scheme.

$\mu\ (mPa\ \cdot s)$	*c* (ppm)	$\lambda\ (s)$	*f* (Hz)	*Wi*
0.97	0	0.0	130	0
1.38	10	2.6×10^{-5}	100	0.03
2.92	35	3.6×10^{-4}	72	0.15
4.51	60	8.98×10^{-4}	66	0.37
6.81	100	2.2×10^{-3}	61	0.85
11.8	225	9.5×10^{-3}	56	3.3
18.8	500	3.96×10^{-2}	54	13.6

Table 3. Viscosity, concentration, relaxation time, and bundle rotation frequencies used to estimate *Wi* in solutions of CMC.

Next, the bundle rotation rates for our polymeric solutions are estimated using their viscosity values. Previous experiments have shown that bundle rotation rate decreases with viscosity [8]. Although the bundle rotation rate is not directly observed in our experiments, the viscosity of the solutions is well characterized (c.f. SI Fig. 1). Hence we can use the available data [8] to estimate the average bundle rotation rate *f*.

Assuming that the bundle rotation rate decreases as the inverse of the viscosity, the estimated bundle rotation rate *f* decreases from 130 to 55 Hz as the polymer concentration increases from 0 to 500 ppm, as shown in Table 3. From these values, our estimated range of $Wi = 2\pi f\lambda$ is 0.03 to 13.6 (Table 3).

B. Weissenberg numbers for DNA polymer near tethered *E. coli*

For the DNA molecule in water, the relaxation time λ is approximately 3 seconds [9]. The estimated shear rates $\dot{\gamma}$ due to the flow generated by the tethered cell (Fig. 6) are approximately 2.8 s^{-1} (SI6), which leads to $Wi = 2\pi f\lambda \approx 8.4$.

SI-Movies

Movie 1

The suppression of cell wobbling is shown in Movie 1, which contains images of a cell in a Newtonian buffer solution and a cell in the polymeric solution at *c* = 225 ppm. Overlaid on the cell body image is the instantaneous body orientation Φ. The wobbling amplitude of the cell in buffer solution is over 20 ℃, while the cell in the polymer solution has amplitude below 5 ℃. These images capture the change in the cells swimming kinematics and the dramatic change in wobbling.

Movie 2

Sample tethered cells are shown in Newtonian fluids (buffer and a solution of PEG) and viscoelastic fluids (solution of CMC). As the viscosity increases, the tethered cells exhibit two changes: (1) a decrease in rotational speed and (2) also a decrease in switching frequency.

Movie 3

Once a tethered cell (dark rod) is identified using bright field microscopy, the polymer molecules (bright blobs) around the cells are visualized with fluorescence microscopy. The polymer molecule can be seen stretching and tend to align with flow.

References:

[1] N.C. Darnton, L. Turner, S. Rojevsky, H.C. Berg, On torque and tumbling in swimming Escherichia coli, *Journal of Bacteriology*, **189,** 1756-1764 (2006).
[2] Y. Sowa, R.M. Berry, Bacterial flagellar motor, *Q. Rev. Biophys.*, **41,** 103-132 (2008).
[3] A.E. Koser, L. Pan, N.C. Keim, P.E. Arratia, Measuring material relaxation and creep recovery in a microfluidic device, *Lab on a Chip*, **13,** 1850-1853 (2013).
[4] X.N. Shen, P.E. Arratia, Undulatory swimming in viscoelastic fluids, *Phys. Rev. Lett.*, **106,** 208101 (2011).
[5] P.S. Lovely, F.W. Dahlquist, Statistical measures of bacterial motility and chemotaxis, *J. theor. Biol.*, **50,** 477-496 (1975).
[6] D.E. Smith, H.P. Babcock, S. Chu, Single-polymer dynamics in steady shear flow, *Science*, **283,** 1724-1727 (1999).
[7] F. Valle, M. Favre, P. De Los Rios, A. Rosa, G. Dietler, Scaling exponents and probability distributions of DNA end-to-end distance, *Phys. Rev. Lett.*, **95,** 158105 (2005).
[8] P.G. de Gennes, Scaling Concepts in Polymer Physics, Cornell University Press, (1979).
[9] T. Su, Entropic elasticity of polymers and their networks, Ph. D. Thesis, University of Pennsylvania: USA, (2011).
[10] T.T. Perkins, S.R. Quake, D.E. Smith, S. Chu, Relaxation of a Single DNA Molecule Observed by Optical Microscopy, *Science*, **264,** 822-826 (1994).

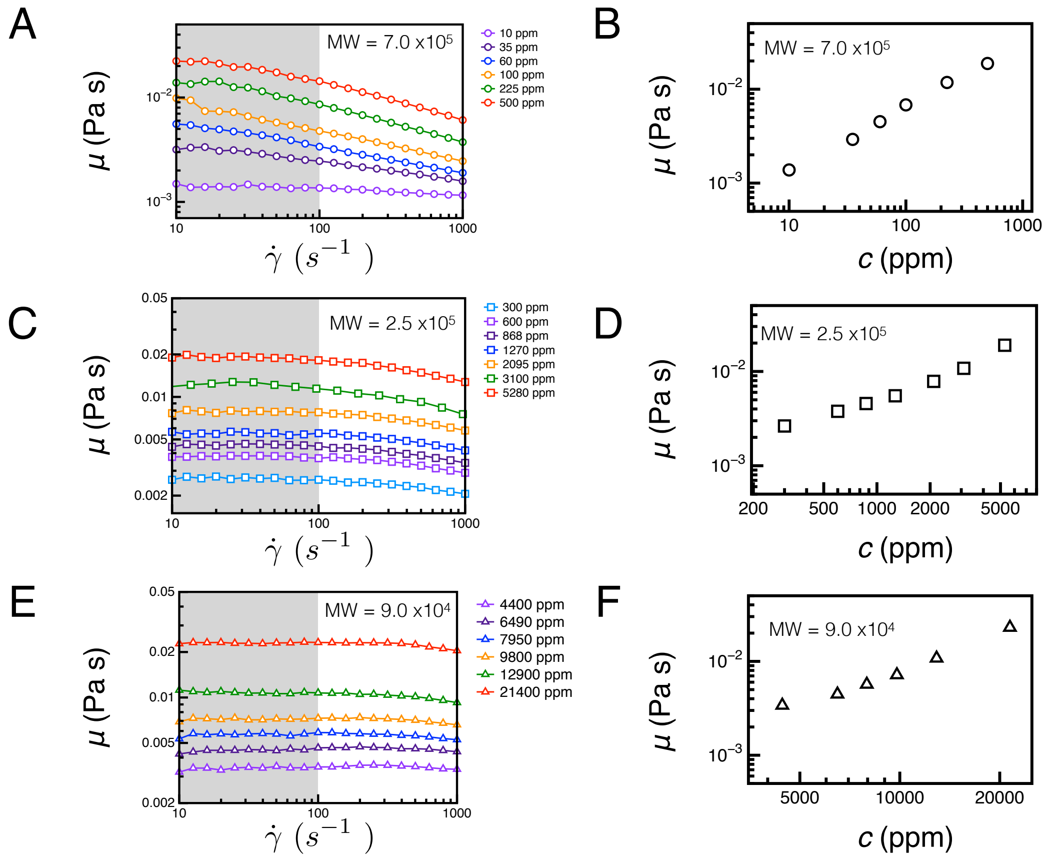

SI-Figure 1: Shear viscosity of CMC solutions. (A) Shear viscosity of CMC (MW = 7.0 x 10^5) at polymer concentrations ranging from 10 to 500 ppm. (B) The shear viscosity magnitude, defined as the mean shear viscosity over 10-100~s^{-1}, increases from 1.0 to 20.0 mPa ·s as the polymer concentration increases. The shear viscosities for MW = 2.5 x 10^5 (C, D) and MW = 9.0 x 10^4 (E,F) also range from 1.0 to 20.0 mPa ·s.

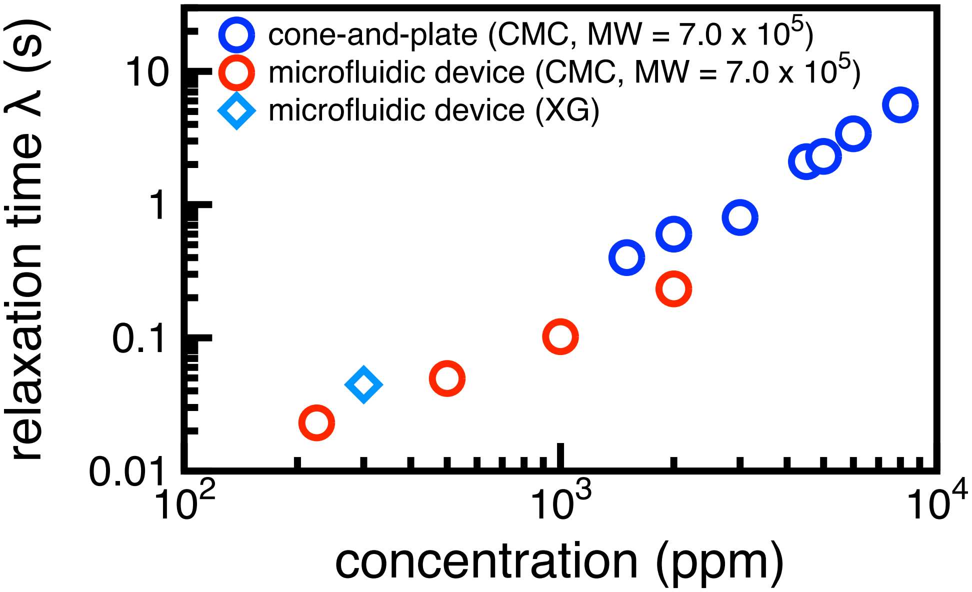

SI-Figure 2: The relaxation times for CMC (MW = 7.0 x 10^5) and XG solutions versus concentration as determined by a microfluidic device are consistent with relaxation time measurements at higher concentrations in a cone-and-plate rheometer [4].

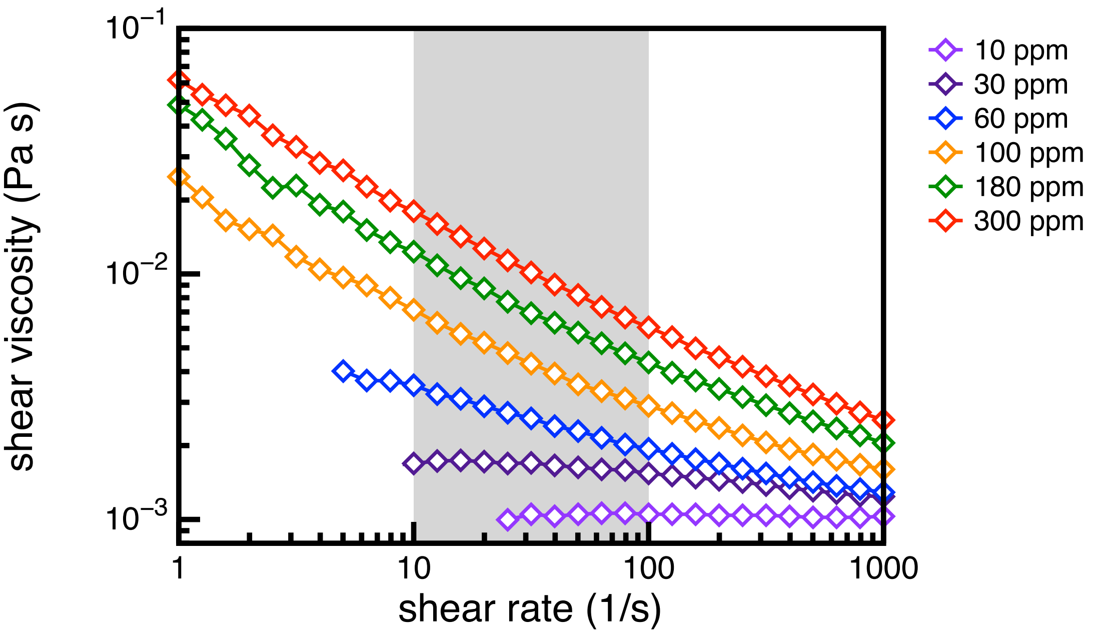

SI-Figure 3: Shear viscosity versus shear rate for Xanthan Gum solutions. The viscosity significantly shear thins, particularly in the region of interest, $\dot{\gamma}$ =10-100 s^{-1}. At the highest concentration, the shear-thinning index *n* reaches a minimum of 0.50.

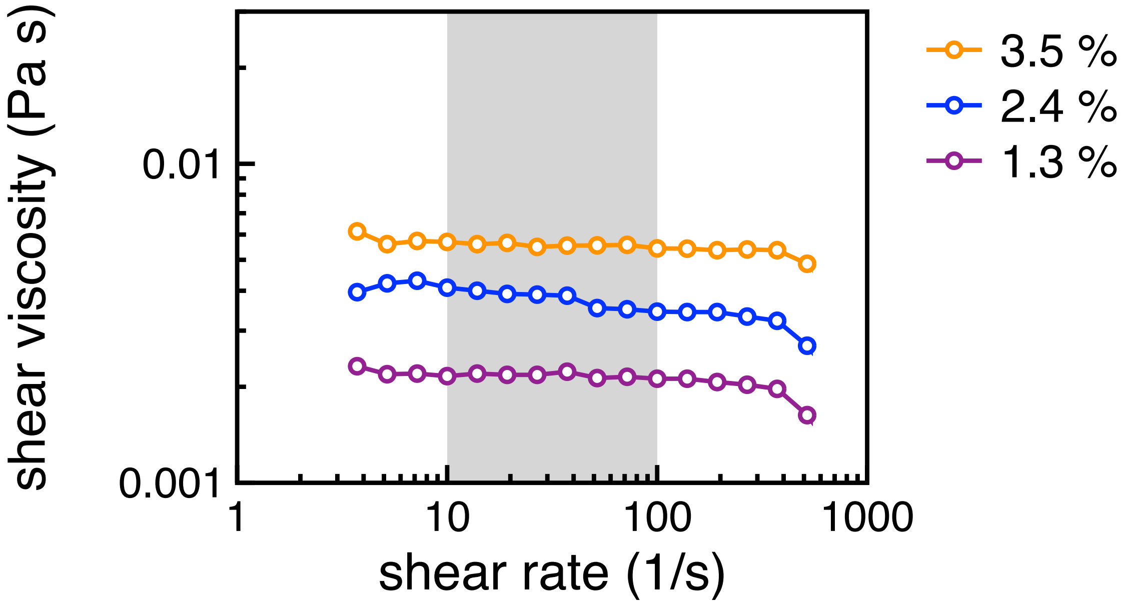

SI-Figure 4: Shear viscosity of PEG solutions at polymer concentrations ranging from 1.3 to 10% by weight. The shear viscosity magnitude increases from 2 mPa·s to 30 mPa·s. There is negligible change in viscosity with shear rate, exhibiting Newtonian behavior.

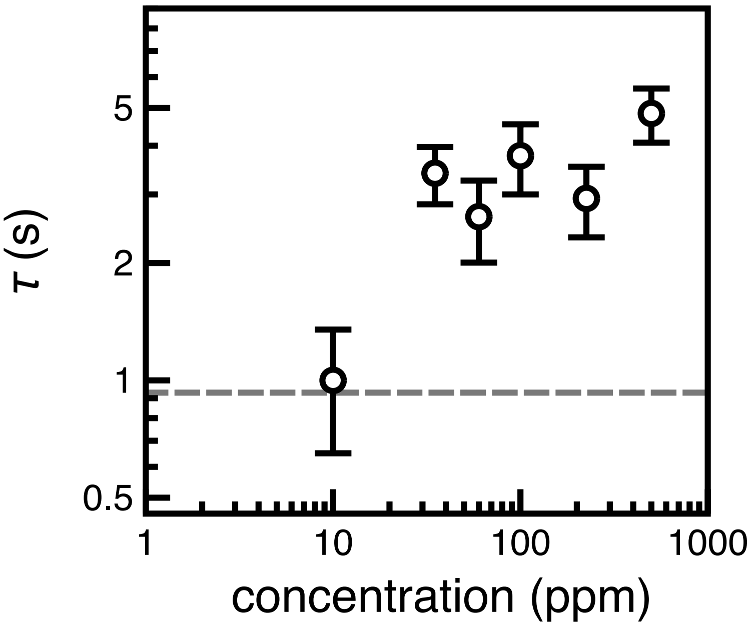

SI-Figure 5: The crossover time τ increases from 0.9 to 4.8 s as polymer concentration increases up to 500 ppm (CMC, MW = 7.0 x 10^5). The result for buffer is included for reference.

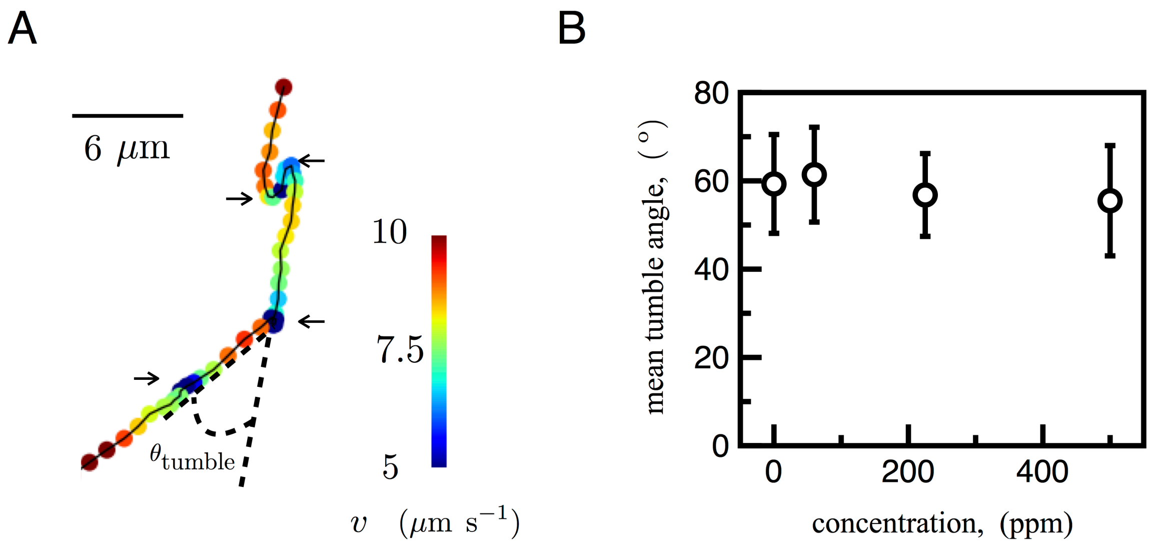

SI-Figure 6: Tumble Angles. (A) The tumble angles are defined by the angular change in direction from run to run. (B) The mean tumble angle, averaged over multiple tumbles in multiple cells, remains nearly constant with concentration.

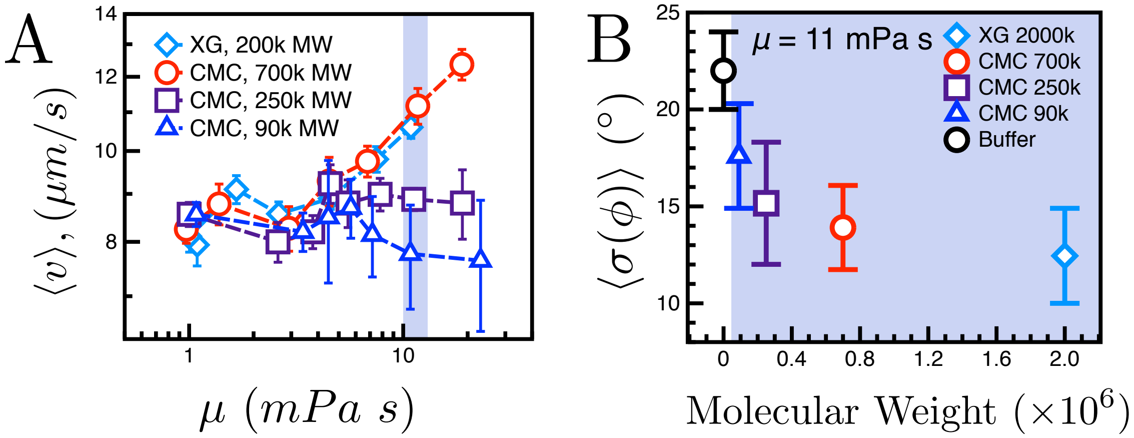

SI-Figure 7: (a) Cell velocities as a function of viscosity for varying polymers. At μ = 11 mPa · s (shaded), the velocity increases with MW. (b) For μ = 11 mPa · s, $\langle \sigma(\phi) \rangle$ decreases with MW, suggesting that fluid elasticity suppresses wobbling. The result in buffer is included for reference.

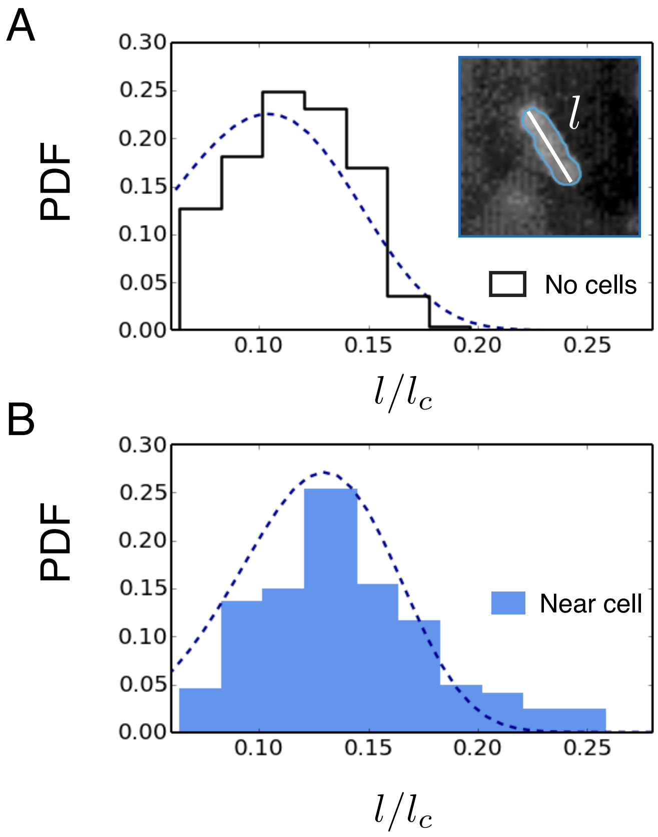

SI-Figure 8: Stretching of polymer molecule quantified using the probability distribution of a normalized extension given by the ratio of the length-scale ℓ (the maximum distance along two points of the contour) and its contour length ℓ_c. (A) The PDF for polymer molecules in the absence of cells. The dashed line corresponds to the predicted PDF for a self-avoiding random walk in two dimensions [7]. (Inset) Definition of the length ℓ. (B) Normalized length fluctuations for the polymer near the cell are due to a contribution of thermal motion and its interaction with the cell. As can be seen the distribution function shifts to the right suggesting an extended state. The dashed line corresponds to equation SI-1.
